# Size Perception Biases Are Temporally Stable and Vary Consistently Between Visual Field Meridians

**DOI:** 10.1177/2041669519878722

**Published:** 2019-09-25

**Authors:** Dietrich S. Schwarzkopf

**Affiliations:** Department of Experimental Psychology, University College London, UK; School of Optometry & Vision Science, University of Auckland, New Zealand

**Keywords:** objects and features, perceptual bias, size perception, spatial heterogeneity

## Abstract

The apparent size of visual stimuli depends on where in the visual field they appear. We recently presented a model of how size perception could be biased by stimulus encoding in retinotopic cortex. However, it remains unclear if such perceptual biases are instead trivially related to discrimination ability and if they are temporally stable. An independent test of the model is also still outstanding. Here, I show that perceptual biases are stable across stimulus durations between 50 and 1,000 milliseconds, even though discrimination ability unsurprisingly improves with duration. Furthermore, perceptual biases are stronger along the vertical than the horizontal meridian, which mirrors reported differences in spatial vision and the positional selectivity of early visual cortex. Taken together, these findings support our model of how size is inferred from cortical responses.

## Introduction

Our impression of a seamless and accurate perception across our visual field belies the fact that the neural representation of the visual field is highly heterogeneous. Spatial visual acuity is highest in central vision and falls off with increasing eccentricity meaning that the visual system encodes only coarse spatial detail in the periphery (Anstis, 1998; Dumoulin & Wandell, 2008). Similarly, visual ability has also been shown to vary considerably between visual field meridians (Anderson, Leslie Cameron, & Levine, 2014; Carrasco, Talgar, & Cameron, 2001). It has also been shown that for many visual functions, there are unique spatial patterns that are reliable but very idiosyncratic in each individual (Afraz, Pashkam, & Cavanagh, 2010; Greenwood, Szinte, Sayim, & Cavanagh, 2017; Kosovicheva & Whitney, 2017; Moutsiana et al., 2016; Schwarzkopf & Rees, 2013; Visconti di Oleggio Castello, Taylor, Cavanagh, & Gobbini, 2018).

We recently formulated a model of how visual object size could be read out from the retinotopic stimulus representation in visual areas (Moutsiana et al., 2016). We argued that the brain could infer size from the cortical separation between activity peaks produced by the object’s edges. The apparent size of stimuli decreases in the periphery (Anstis, 1998; Bedell & Johnson, 1984; Newsome, 1972), where its spatial location is encoded less precisely in retinotopic cortex, as measured by population receptive field (pRF) spread (Dumoulin & Wandell, 2008; Moutsiana et al., 2016; Smith, Singh, Williams, & Greenlee, 2001). We showed that idiosyncratic spatial patterns in pRF spread correlate with the spatial heterogeneity of size perception biases across the visual field. Critically, this model could explain both decreases and increases in apparent size under different stimulus conditions (Moutsiana et al., 2016).

We are normally unaware of such perceptual biases. Do they only manifest when stimuli are flashed briefly? Moreover, if observers move their eyes during longer stimulus presentations and foveate the stimuli, does this reduce their perceptual biases? It also remains unclear whether these perceptual biases are not simply due to other trivial factors unrelated to the model, such as poorer discrimination ability or impoverished stimulus information (Bedell & Johnson, 1984; Newsome, 1972). It is also possible that longer stimulus presentations provide observers with more opportunity to adjust their decision cognitively and thus correct for their perceptual biases.

Therefore, in my first experiment, I tested whether these size perception biases depend on stimulus duration. Observers performed the Multiple Alternatives Perceptual Search (MAPS) task to measure perceptual biases at four parafoveal locations (Finlayson, Manser-Smith, Balraj, de Haas, & Schwarzkopf, 2018; Finlayson, Papageorgiou, & Schwarzkopf, 2017; Moutsiana et al., 2016). This entails reporting which of four candidate stimuli perceptually matches the size of a constant reference stimulus shown at fixation. Unlike in previous studies in which the stimuli were only shown for a brief 200 milliseconds, I varied the stimulus duration from 50 to 1,000 milliseconds and quantified whether perceptual biases change with duration.

Importantly, our previous model was purely descriptive. A critical confirmation of this model must test the predictions it makes for size perception on *new* data under conditions where pRF spread should vary consistently. In my second experiment, I therefore tested a crucial prediction of our model that stimuli should be perceived as smaller (more biased) in locations where the spatial encoding in corresponding parts of visual cortex is poorer (Moutsiana et al., 2016). Recently, Silva et al. (2017) suggested that pRF spread is broader along the vertical than the horizontal meridian, which ties in with suggestions of poorer spatial vision along the vertical meridian (Anderson et al., 2014; Carrasco et al., 2001). Our model therefore predicts that objects should be perceived as smaller (stronger perceptual bias) along the vertical than the horizontal meridian.

## Methods

### Participants

Twenty-one observers (ages 19–38 years, 13 females, 4 left-handed) participated in Experiment 1, including the author. The design of Experiment 2 was preregistered (see osf.io/8u2z5). Thirteen observers (ages 20–39 years, 8 females, 2 left-handed, 1 ambidextrous) participated in Experiment 2 using a Bayesian sampling plan (see below for details). The author also participated, but his results were excluded from the inferential statistical analysis because his data were acquired before preregistration. All observers gave written informed consent, and procedures were approved by the University College London Research Ethics Committee. All observers had normal or corrected-to-normal visual acuity. In Experiment 2, there was a predefined exclusion criterion that any observer whose accuracy on the MAPS task for an experimental run was 30% or less would be excluded (note that chance performance is 25%). All observers performed the task above criterion on all runs and therefore nobody was excluded.

### Stimuli

Observers were presented with a stimulus array containing four light gray, parafoveally presented circle stimuli (the candidates) and one reference circle shown in the center of gaze. The background was black. A blue fixation dot (0.2° visual angle) was also present in the center of gaze. The sizes of three of the candidates relative to the size of the reference (0.98° visual angle) were drawn from a Gaussian distribution (μ = 0, σ = 0.3) expressed in binary logarithmic units. The size of the fourth candidate was identical to the size of the reference. These stimuli have been described in more detail previously (Finlayson et al., 2017, 2018; Moutsiana et al., 2016).

In Experiment 1, the candidates were presented along the oblique axis in each visual field quadrant at 3.92° eccentricity. Stimulus duration was 50 milliseconds, 100 milliseconds, 200 milliseconds, 500 milliseconds, or 1,000 milliseconds, pseudo-randomly interleaved across trials. The experiment took approximately 30 minutes per observer.

In Experiment 2, candidates were presented along the vertical and horizontal meridians at an eccentricity of 3.92° or 7.84°, the middle and outer eccentricity we had previously used (Moutsiana et al., 2016). (In the preregistration document, this was incorrectly defined as 7.94°.) Stimulus duration was always 200 milliseconds. The experiment took 16 to 25 minutes per observer.

### Procedure

In both experiments, observers fixated a central dot and performed the MAPS task (Finlayson et al., 2017, 2018; Moutsiana et al., 2016). Observers were instructed to select the candidate that appeared most similar in size to the reference using keyboard buttons corresponding to the four locations. Following their choice, a ripple effect indicated the chosen location and the fixation dot briefly changed by increasing its size to 0.33° for 50 milliseconds. No feedback about the correctness of the response was given, which differs from most of our previous experiments using the MAPS task. We recently showed that perceptual bias estimates are greater without feedback even though spatial patterns of bases are similar irrespective of whether feedback is given (Finlayson et al., 2018). However, in both experiments, most participants were given the opportunity to briefly familiarize themselves with the task before the actual experiment commenced. During these practice trials, feedback was given by turning the fixation dot green for 50 milliseconds if they had picked the correct target on a trial.

In Experiment 1 only, the observers’ eye movements were binocularly recorded at 60 Hz using a Tobii EyeX desk-based eye tracker running custom binding code by Pete Jones (https://www.ucl.ac.uk/∼smgxprj/resources.html), calibrated prior to the experiment. There were normally 1,000 trials in total in Experiment 1 and 200 trials per stimulus duration. Every 20 trials, observers were given a brief rest break and asked to continue by pressing any button on the keyboard. Already acquired data were saved at each rest block. Due to an unresolvable technical issue with the eye tracking code, the protocol sometimes crashed. When that happened, the experiment was restarted, and the number of still required blocks reduced accordingly. Thus, some participants performed a small number of additional unrecorded trials. As previously (Finlayson et al., 2017, 2018; Moutsiana et al., 2016), the buttons responding on each trial were F, V, M, and K corresponding to the four visual field quadrants.

In Experiment 2, there were 400 trials per run and observers performed two runs, one per eccentricity. The order of eccentricity conditions was pseudo-randomized for each observer. There was a rest break every 20 trials. The buttons for making behavioral responses were the four arrow buttons denoting the candidate above, below, left, or right of fixation, respectively.

### Data Analysis

MAPS fits a model to the behavioral responses to quantify the perceptual bias, the size an observer required to perceptually match the candidate at a given location to the reference, and the discrimination ability, the uncertainty with which the observer chose a candidate at that location. The model contains a Gaussian similarity detection function at each candidate location, where the peak location reflects the perceptual bias, and the standard deviation denotes uncertainty. For each trial, the model calculates the output of the similarity detector given the current stimulus at a given location. It then predicts that the observer chose the location where this output was maximal. The four bias and uncertainty parameters are fit by maximizing the prediction of the observer’s actual behavioral responses across all trials (see Finlayson et al., 2017, 2018; Moutsiana et al., 2016, for more details).

In Experiment 1, I quantified how perceptual biases and discrimination performance depended on stimulus duration. I averaged parameter estimates across the four candidate locations because here I was only interested in the magnitude of these measures rather than their spatial patterns. I also quantified the eye position in each trial and analyzed this separately for each duration condition. I removed artifacts by deleting empty samples and any samples for which the horizontal or vertical eye position was further than three standard deviations from the mean. I then calculated the variance for the horizontal and vertical positions and then converted this into a Euclidean distance (square root of the sum of the squares of these variances). Finally, I removed participants for whom this measure exceeded 4° (or 2°) visual angle as these constituted excessively noisy recordings.

In Experiment 2, I compared perceptual biases and discrimination ability between the vertical and horizontal meridian. I averaged parameter estimates across all locations on each meridian irrespective of eccentricity or visual hemifield (i.e., upper and lower hemifield for vertical meridian, left and right hemifield for horizontal meridian). I collected data from 10 observers and then continued sampling until a Bayesian paired *t* test (Rouder, 2014; Rouder, Speckman, Sun, Morey, & Iverson, 2009) comparing perceptual biases for the vertical and horizontal meridians favored either the alternative or the null hypothesis. The default prior had a scale factor of 0.707, and the stopping criterion was a Bayes factor (BF) >10 or <0.1. The scale of the default prior was chosen in accordance with plausible effect sizes in psychological research, but the results presented are qualitatively unaffected by the exact choice. I set an upper maximum sample size of *n* = 30, but the stopping criterion was already reached at *n* = 13. I also compared biases between the various subconditions and also conducted the same analyses for discrimination ability (uncertainty).

## Results

### Experiment 1

**Figure 1.**
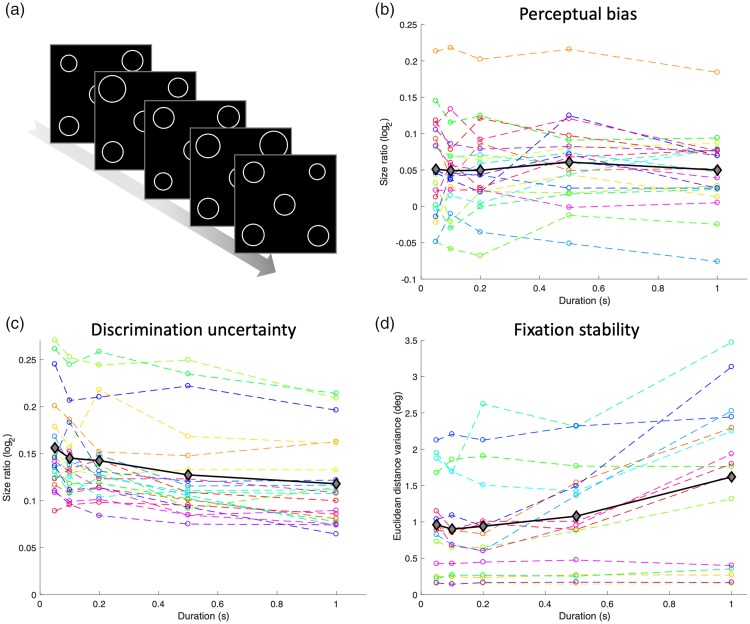
(a) Schematic illustration of a sequence of trials in the MAPS task (Moutsiana et al., 2016) in Experiment 1. In each trial, observers were shown an array of circles and instructed to select the quadrant with the candidate circle that best matched the size of the central reference. Perceptual bias estimates (b), discrimination uncertainty (c), and fixation stability (d) plotted against stimulus duration. Dashed lines in colors denote individual observers. The solid black lines with diamond symbols denote the group mean.

Using MAPS (Figure 1(a)), I tested how perceptual biases and discrimination ability depended on stimulus duration. Mean bias was stable regardless of stimulus duration (Figure 1(b)) with no significant difference between different durations, one-way repeated-measures analysis of variance: *F*(4, 80) = 1.03, *p* = .397, BF_10_ = 0.004; converting *F* ratios to BFs (Faulkenberry, 2018). However, discrimination ability (uncertainty; Figure 1(c)), was significantly better at longer stimulus durations, *F*(4, 80) = 26.84, *p* < .001, BF_10_ = 17.2.

To quantify fixation stability, I calculated for each duration the variance of the Euclidean distance from fixation, thus combining horizontal and vertical eye position. Because eye tracking sometimes failed or produced artifactual deviations (>4°), the data from four participants were excluded. While a small number of observers maintained stable fixation irrespective of duration, on average fixation was significantly less stable, *F*(4, 60) = 12.76, *p* < .001, at longer durations (Figure 1(d)), although Bayesian inference only showed inconclusive evidence (BF_10_ = 0.538). Critically, even when using a more stringent criterion (eye deviations <2°), perceptual biases were constant across durations, *F*(4, 36) = .98, *p* = .429, BF_10_ = 0.017. When excluding three observers whose overall performance was ≤30%, results were also qualitatively unchanged, bias: *F*(4, 68) = 0.85, *p* = .498, BF_10_ = 0.005; uncertainty: *F*(4, 68) = 21.25, *p* < .001, BF_10_ = 4.6; fixation: *F*(4, 68) = 3.69, *p* = .009, BF_10_ = 0.018.

To further explore whether the rate with which fixation stability worsened with duration could predict the change in perceptual biases, for each observer I fit a linear regression between duration and fixation stability or perceptual bias, respectively. The regression coefficients for these parameters were uncorrelated (slope: *r* = −0.18, *p* = .515, BF_10_ = 0.234; intercept: *r* = −0.05, *p* = .852, BF_10_ = 0.192).

In summary, I found no effect of stimulus duration on perceptual biases. However, discrimination ability improved at longer durations while fixation was less stable.

### Experiment 2

**Figure 2.**
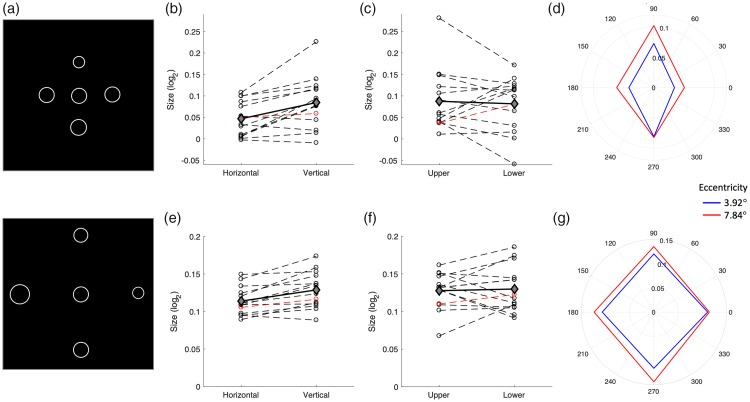
(a) In Experiment 2, candidate stimuli were presented on the vertical and horizontal meridian (above, below, left, and right of the central reference) at two different eccentricities (upper and lower panel). Perceptual bias (b to d) and discrimination uncertainty (e to g) estimates for the horizontal versus vertical meridian (b and e), the upper versus lower visual field of the vertical meridian only (c and f), and separately for each tested location (d and g). Dashed lines in (b), (c), (e), and (f) denote individual observers. Solid black lines show the group mean. The red dashed line corresponds to the author’s data which were excluded from statistical inference. In (d) and (g), data are separated by eccentricity (blue: 3.92°; red: 7.84°).

I next tested if perceptual biases were stronger on the vertical than horizontal meridian (Figure 2(a)) as our model (Moutsiana et al., 2016) would predict based on a recent report that pRF spread is greater on the vertical meridian (Silva et al., 2017). Using a Bayesian sampling plan (Rouder, 2014), I collected data until the BF on a paired *t* test comparing mean biases for the two meridians favored either the alternative or null hypothesis at a ratio of 10:1. The evidence clearly supported the alternative hypothesis, *t*(12) = −3.61, *p* = .004, BF_10_ = 13.5, as the mean biases along the vertical meridian were almost twice as strong as those on the horizontal meridian (Figure 2(b)). Because a positive bias reflects how much a stimulus must be enlarged to be perceptually matched to the reference stimulus, the more positive the bias estimate the *smaller* the apparent stimulus size.

Separating data by eccentricity confirmed that this difference manifested both at 3.92° eccentricity, *t*(12) = −4.64, *p* <.001, BF_10_ = 64.2, and at 7.84° eccentricity, although the latter effect was less robust, *t*(12) = −2.52, *p* = .027, BF_10_ = 2.6. Finally, I also tested whether biases along the vertical meridian differed between the upper and lower hemifields (Figure 2(c)). Here, I found no significant difference and statistical evidence instead weakly favored the null hypothesis, *t*(12) = 0.58, *p* = .570, BF_10_ = 0.323.

Next, I also conducted the same comparisons for the discrimination ability, as quantified by the uncertainty parameter in MAPS. Again, I found a significant difference between meridians (Figure 2(e)), with performance being better for the horizontal than the vertical meridian, *t*(12) = −4.12, *p* = .001, BF_10_ = 29.5. This difference was also significant for the outer eccentricity of 7.84°, *t*(12) = −4.69, *p* < .001, BF_10_ = 69.3, but not for the inner eccentricity of 3.92°, *t*(12) = −1.59, *p* = .137, BF_10_ = 0.766. As for perceptual biases, there was no significant difference in uncertainty between the upper and lower visual field on the vertical meridian (Figure 2(f)), but rather results weakly favored the null hypothesis, *t*(12) = −0.19, *p* = .853, BF_10_ = 0.283.

The polar plots in Figure 2(d) and (g) illustrate the mean perceptual biases and uncertainties across the group separately for each visual field position.

## Discussion

In two experiments, I addressed questions about size perception biases as measured by the MAPS task. Observers judged which of four candidate stimuli presented at parafoveal eccentricities best matched the size of a centrally presented reference. I then quantified the perceptual bias, at which size a candidate stimulus appeared the same as the reference, and the discrimination ability, how uncertain observers were in making their perceptual decisions.

In the first experiment, I found that perceptual bias, a systematic underestimation of parafoveal stimulus size similar to that reported in our previous studies, was constant irrespective of stimulus duration. Thus, even when observers have more time to view the stimuli, they still misperceive the size of stimuli.

Interestingly, discrimination ability, as quantified by the uncertainty parameter in MAPS, improved with longer stimulus durations. It perhaps stands to reason that the task becomes easier at longer durations. Neurons encoding the stimulus will fire for a longer time period at longer stimulus presentations and thus the visual system can accumulate more information, resulting in more reliable estimates of its position and size. This, however, does not reduce perceptual bias.

Perhaps also unsurprisingly, fixation stability became worse at longer stimulus durations. A brief 50-millisecond stimulus presentation is too short to allow voluntary eye movements, but at the longest duration, 1,000 milliseconds, participants may have saccaded toward the parafoveal candidate stimuli and microsaccades and eye movement jitter could have occurred. Worse fixation compliance for longer durations could also partly explain why discrimination ability increased at longer durations.

Critically, however, even though observers tended to make more eye movements during longer stimulus presentations, their perceptual biases remained unaffected. Theoretically, if observers foveated the individual stimuli in turn (challenging even at a duration of 1,000 milliseconds), their *biases should have decreased with duration*. Yet the rate with which fixation stability changed with duration did not predict the change in perceptual bias.

Taken together, my results therefore suggest trivial factors like eye movements, stimulus duration, or discrimination ability cannot explain these perceptual biases. In the second experiment, I then tested a critical prediction by our previous model (Moutsiana et al., 2016): Perceptual biases should be stronger along the vertical than the horizontal meridian. This was based on a recent finding that spatial encoding in early visual areas, as quantified by pRF spread, is broader along the vertical meridian (Silva et al., 2017). I confirmed this prediction in a preregistered design. At two eccentricities, perceptual biases were indeed more pronounced—stimuli were perceived as smaller—when they were presented on the vertical than the horizontal meridian. Moreover, discrimination ability was better along the horizontal meridian, which is also consistent with smaller pRFs (Silva et al., 2017) and more accurate spatial vision along the horizontal meridian (Anderson et al., 2014; Carrasco et al., 2001).

I also conducted a secondary comparison of perceptual biases and discrimination ability between the upper and lower visual field. Previous work had suggested such hemifield differences for pRF spread (Silva et al., 2017); however, I found no evidence of such differences in terms of perception. This could be due to the fact that the earlier pRF differences between hemifields were far less pronounced than differences between the meridians. Naturally, I also had less data for this comparison because there were only half the number of measurements and therefore statistical power for this comparison was lower. There could also be considerable interindividual variability in the functional organization of the hemifields.

Thus, the pattern of perceptual biases along the meridians supports our model of how the visual system infers object size from representations in early visual cortex (Moutsiana et al., 2016). Responses are presumably sustained over the duration of stimulus presentation and so the model predicts that perceptual biases remain constant also. The fact that unstable fixation does not reduce biases could indicate that only the initial response to the stimulus determines observers’ biases. This is, however, inconsistent with the fact that discrimination ability improves at longer stimulus durations, even though bias does not.

In conclusion, my results rule out trivial alternative explanations for how these perceptual biases arise and support predictions made by our basic readout model of visual size perception (Moutsiana et al., 2016).

## Data Accessibility

Data and materials are available for download (Experiment 1: osf.io/32jqu and Experiment 2: osf.io/au78z).
